# 2004–2017 Geospatial dataset of wild and prescribed fire activity over the conterminous United States

**DOI:** 10.1016/j.dib.2024.110856

**Published:** 2024-08-19

**Authors:** James Beidler, George Pouliot, Kristen Foley

**Affiliations:** US Environmental Protection Agency, 109 T.W. Alexander Drive, Research Triangle Park, NC 27711, United States

**Keywords:** Wildland fires, Fire events, Fire timeseries, Fire type

## Abstract

Wildland fire activity is provided in a geospatial database of polygons over the conterminous United States for the 2004 through 2017 time period. The location, timing, and size of the fires are derived from a fusion of wildland fire activity from a consistent set of national ground reports, satellite, and geospatial fire data. A combination of information from the underlying data sources and a regional climatological approach is used to differentiate prescribed fire from unplanned wildfire. The data were developed as part of the United States Environmental Protection Agency (US EPA) Air Quality Time Series project (EQUATES). This dataset can be useful for the evaluation of wildland fire activity over time and across regions.

Specifications Table

**Table:** 

Subject	Environmental sciences, forestry.
Specific subject area	Wildland fire activity
Type of data	Geospatial, file geodatabase
Data collection	This dataset was generated using software and third-party data sources.
Data source location	U.S. Environmental Protection Agency, Research Triangle Park, NC, USA
Data accessibility	Repository name: CMAS Data Warehouse Data identification number: https://doi.org/10.15139/S3/PCXTMU Direct URL to data: https://dataverse.unc.edu/dataset.xhtml?persistentId=doi:10.15139/S3/PCXTMU

## 1 Value of The Data

• This dataset provides a consistent multiyear geospatial dataset of both wild and prescribed fires that can be used for fire activity trends analysis by type for the conterminous United States.
• The dataset can be used for geospatial analysis when coupled with other datasets such as wildland-urban interface perimeters for fire risk managers or landcover for ecologists.
• Identifiers within the dataset can provide linkage to air quality emissions in the EQUATES time series project for modeling and analysis of air quality, including air quality related human health effects.

## 2 Background

The EPA EQUATES time series dataset [1] contains wildland fire locations and air quality emissions differentiated by fire type but lacks corresponding spatially specific wildland fire information that could be used in geospatial analyses. This geospatial dataset was initially built as a bridge between the disparate U.S. fire activity data sources used in the EQUATES fused fire activity estimation methodology and the air quality emissions estimates.

## 3 Data Description

**Fig. 1:**
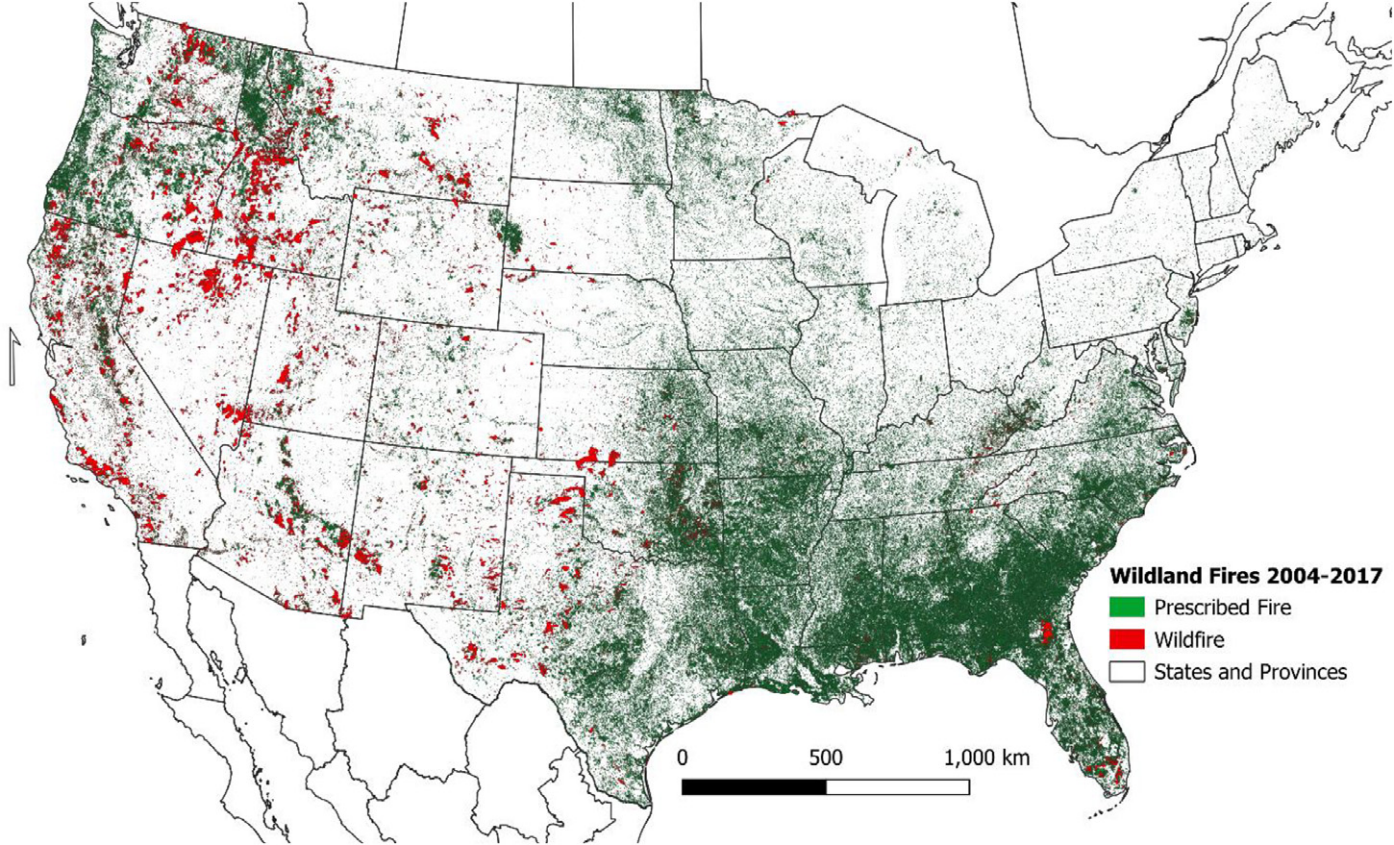
Geospatial extent of the 2004-2017 wildland fire events.

The geospatial database described in this article includes wildland fire events in the conterminous United States (CONUS) from 2004-2017 differentiated by fire type (Fig. 1). The file geodatabase contains event polygons on a Conus Albers projection (EPSG: 5070).

Nine fields are used to describe each fire event in the geodatabase (Table 1). Events with a *fire_type* of RX indicate a prescribed fire, while WF indicates a wildfire. Forty-five fires in 2016 and 2017, have a fire type of PB, indicating a pile burn. The *display_*name field contains the name of the fire event as a string of up to 80 characters. Fires with a *display_name* of “Unknown Fire” indicate an event that is identified only using remotely sensed fire activity data. The unique fire event identifier, *id*, is an integer value from 1 - 999,999 that can be used to associate the polygons with the EQUATES project wildland fire emissions annual flat file inventories *facility_id* to retrieve estimates of wildland fire air emissions. The corresponding EQUATES annual flat file emissions inventories developed for the SMOKE model [2] are not part of this dataset but may be obtained from the EQUATES repository [1]. Example fire geodatabase field values are shown in Table 2.

**Table 1:** Description of the fields provided for each event in the 2004–2017 wildland fire geodatabase.

Field ID	Field Name	Field Type	Valid Range	Description
1	id	integer	0-999999	Unique wildland fire event identifier
2	display_name	string	1 to 80 characters	Name of wildland fire event if designated by underlying dataset
3	end_date	date	1/1/2004 - 12/31/2017	Date of last recorded fire activity for the wildland fire event
4	confidence	float	0-1	Confidence value between 0 and 1 that a fire event occurred as presented based on the underlying datasets, higher numbers indicate that the event was more likely to have occurred as described
5	start_date	date	1/1/2004 - 12/31/2017	Date of ignition or first report of fire activity of the wildland fire event
6	fire_type	string	RX, WF, PB	Identified fire type, possible values include prescribed fire (RX), wildfire (WF), and prescribed burn (PB)
7	year	integer	2004-2017	Year of event ignition derived from the start_date field
8	acres	float	0.0001 - 1,000,000	Total area in acres of the wildland fire event
9	state	string	AL-WY	Two-character US postal code indicating the state with the largest overlap of the event by area

**Table 2:** Example wildland fire event field values from geodatabase.

id	display_name	end_date	confidence	start_date	fire_type	year	acres	state
382606	Oregon Gulch Fire	8/14/2014	0.9999996	7/30/2014	WF	2014	35200	OR
365914	Unknown Fire	1/7/2012	0.9	1/7/2012	RX	2012	99.99	MN
646876	Sesnon Fire	10/22/2007	0.998	10/21/2007	WF	2007	32.45	CA

**Fig. 2:**
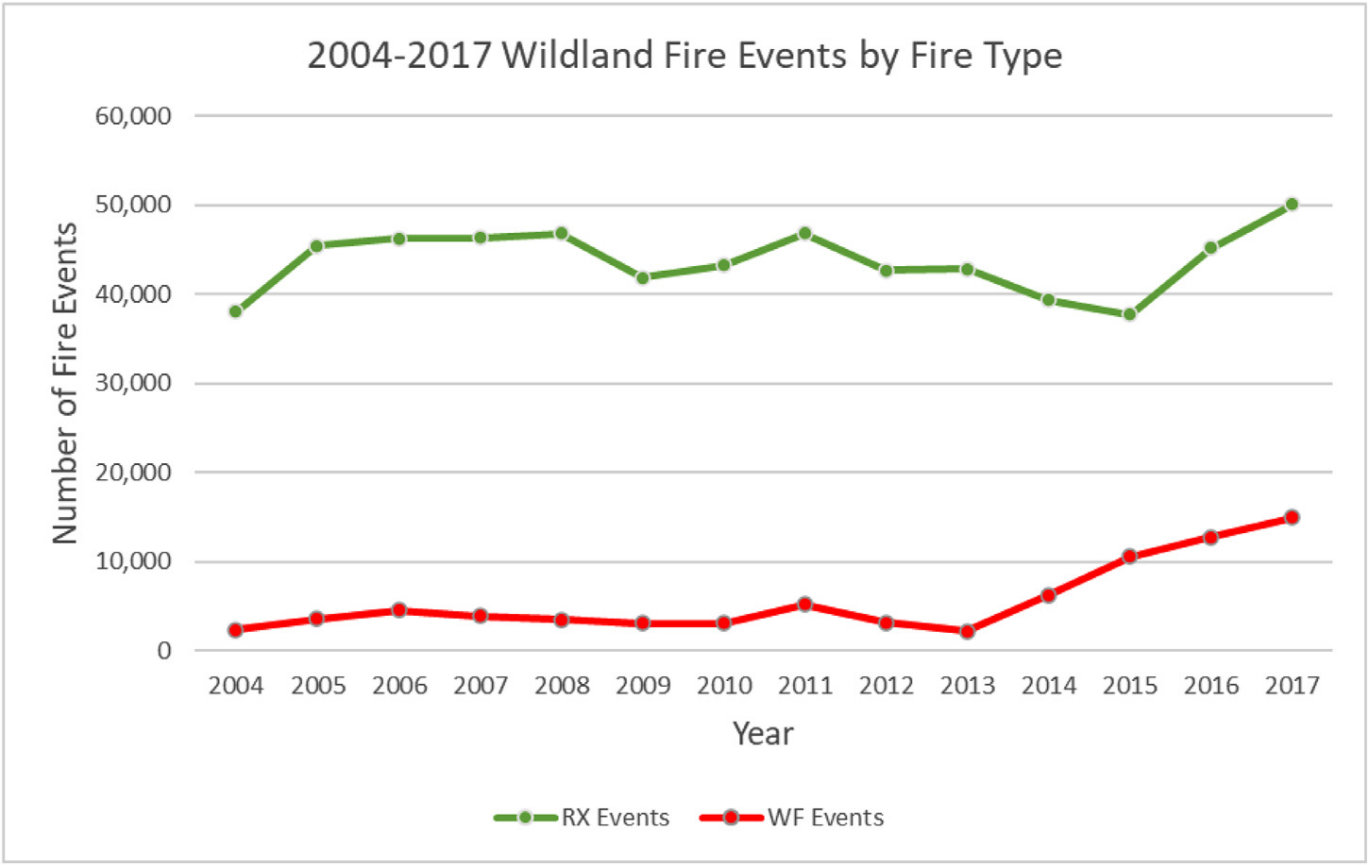
Annual prescribed (RX) and wildfire (WF) wildland fire events in the geodatabase.

**Fig. 3:**
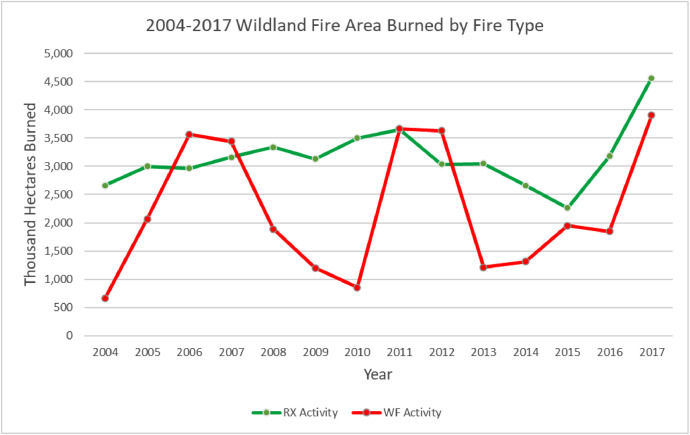
Annual prescribed (RX) and wildfire (WF) wildland fire events estimated hectares burned.

**Fig. 4:**
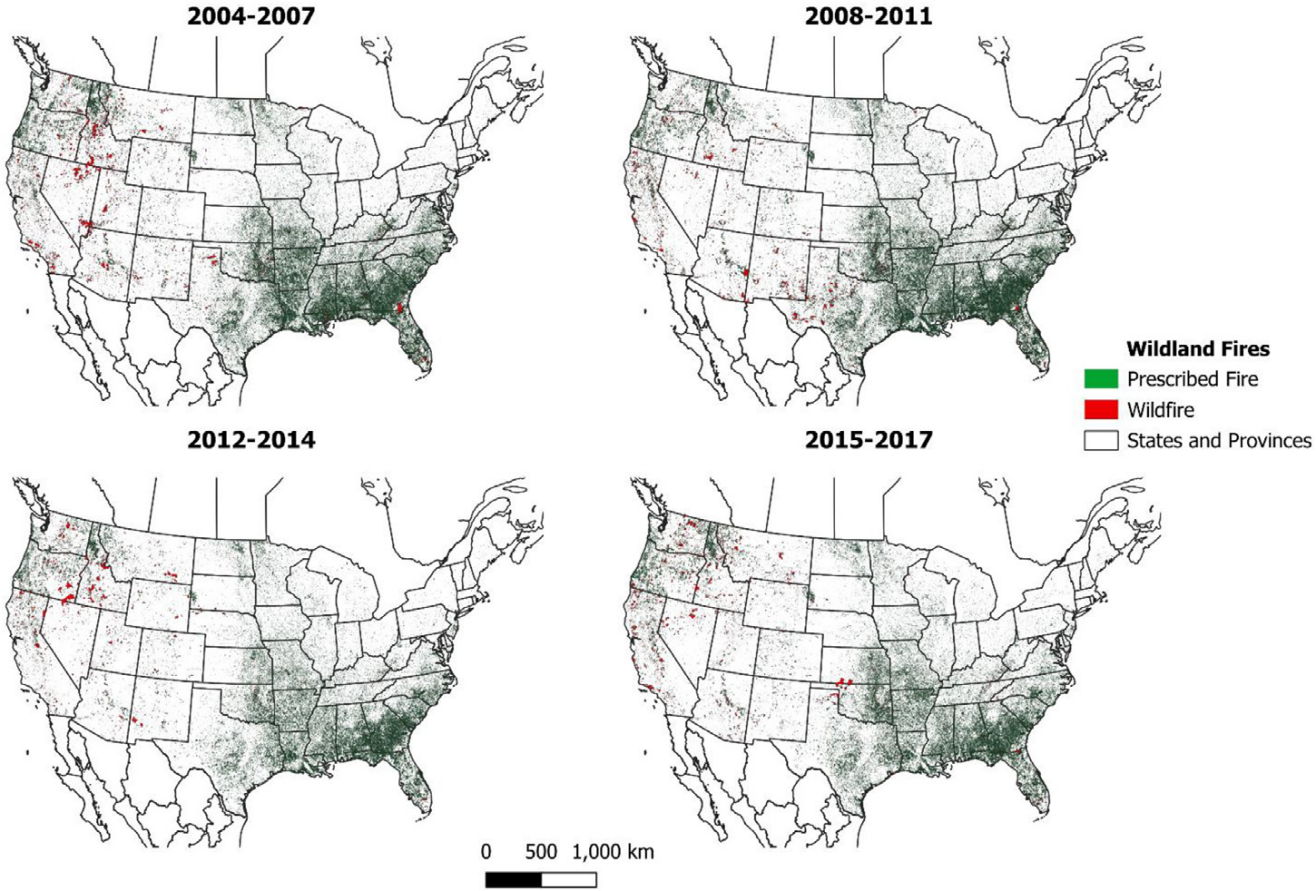
Spatial variation of wildland fire events within the extents of the dataset.

The wildland fire event geodatabase contains 692,268 unique wildland fire events [Fig. 2] that account for an estimated 75.37M hectares burned [Fig. 3]. The locations and size of the events vary annually [Fig. 4].

## 4 Experimental Design, Materials and Methods

### 4.1 Description of Input Wildland Fire Activity Data

The 2004-2017 wildland fire event activity was developed with national fire activity datasets fused using the Satellite Mapping Automated Reanalysis Tool for Fire Incident Reconciliation version 2.0 (SF2) [3]. Source code for SF2 is available from Airfire on github (https://github.com/pnwairfire/SmartFire2). Four activity datasets representing satellite, ground, and perimeter activity were extracted to use in the SF2 fire activity reconciliation process.

Day-specific remote sensed satellite fire data from the Hazard Mapping System (HMS) dataset [4,5] served as the primary source of activity for fire location, timing, and growth. The HMS data also provides a source of wildland fire activity for smaller events not reported in national datasets, such as those that occur on private lands. Daily HMS text files containing the time, location, and sensor information were retrieved for all years from the NOAA website (https://satepsanone.nesdis.noaa.gov/pub/FIRE/web/HMS/Fire_Points/Text/). The United States Department of Agriculture Cropland Data Layer (CDL) [6] was used as a geospatial filter to split out HMS detections over cropland from the wildland dataset to constrain the selection to wildland fires. The HMS dataset contains information from multiple satellites that may result in duplicate records for a daily fire burn location. To avoid the duplication of burned area, the HMS detects over wildland regions were further subset to remove duplicates based on daily latitude and longitude location rounded to three decimal degrees, approximately half of the nominal pixel width of the highest resolution sensor. The Flint Hills grassland region of Kansas and Oklahoma is broadly treated with prescribed fire between February and May every year. The exact date range of prescribed fire activity and the counties where the burns occur in the Flint Hills varies by year. Kansas provides an annual Flint Hills prescribed fire acres burned report on the Kansas State Flint Hills website (https://www.ksfire.org/) [7] that is used to identify the date range and counties of the burns. To obtain a more accurate estimation of the total area burned in this region, HMS detects that fall within the Flint Hills for the annual burn date range are split from the activity used in reconciliation for this dataset for separate processing.

Geospatial Multi-Agency Coordination (GeoMAC) active fire perimeters [8] provided an annual dataset of high geospatial resolution wildfire perimeters in the United States. These wildland fire perimeters include active WF events of all size on federal, state, and private lands with updates over the duration of the fire. This dataset was downloaded as a shapefile for all years 2000–2018 (https://data-nifc.opendata.arcgis.com/datasets/nifc::historic-perimeters-combined-2000-2018-geomac/explore). Polygons associated with fire events for years 2004–2017 were extracted into year-specific shapefiles to reduce dataset size and computational load during processing. GeoMAC perimeter polygons with missing date information or incomplete geometries were removed because they lacked all required information for a wildland fire event (Table 1). For WFs with multiple incident responders and for those WFs lasting more than one day multiple fire perimeters may be created for an individual event. To avoid geospatial processing issues and double counting, the polygon area was calculated in QGIS [9] using the geometry area attribute. A polygon was flagged for potential removal if it exactly matched the area or was smaller than a preceding polygon associated with the same unique fire identifier. These flagged polygons were dropped where perimeter polygons were visually verified as an exact geometric duplicate. The fix geometries tool in QGIS was run to repair any topology issues that may cause problems in the SF2 processing.

The Monitoring Trends in Burn Severity (MTBS) [9] burned areas boundaries shapefile provided an additional high geospatial resolution dataset of large wild and prescribed fires in the United States. MTBS contains remotely sensed burn scar perimeters for both WF and RX fire types with an event area over 404.686 hectares in the western United States and over 202.343 hectares in the eastern United States. The MTBS burned area boundaries dataset was retrieved as a single geospatial file containing all available years (https://edcintl.cr.usgs.gov/downloads/sciweb1/shared/MTBS_Fire/data/composite_data/burned_area_extent_shapefile/mtbs_perimeter_data.zip). Polygons associated with burn scars from years 2004–2017 were extracted into year-specific shapefiles to reduce dataset size and computational load during SF2 processing. While a fire type is given in MTBS it is derived from geospatial association with fires in other datasets. This way of matching fire type results in an incomplete characterization of burn scar fire type. Burn scars without a fire type defined in MTBS were assigned a fire type of UNK and removed from processing because they did not contain all necessary fields. The fix geometries tool in QGIS was run to repair geospatial topology issues that may cause issues in SF2 processing.

Fire incident situation burn reports from the annual Incident Status Summary (ICS-209) situation summary database (SIT-209) [10] contain ground reports of small to large wildland fires. Each fire event record contains a location, start date, end date, and total burned area. The SIT-209 datasets were retrieved as self-extracting ACCESS formatted databases specific to each year from the Wildland Fire Application Information Portal (https://www.wildfire.gov/application/sit209). The SIT-209 databases include records for both fire incidents and non-fire incident data such as tornado or other disaster responses. Only those records with an incident type code indicating a wildland fire were kept from the SIT209_HISTORY_INCIDENT_209_REPORTS and SIT209_HISTORY_INCIDENTS tables of the annual summary database and exported into Excel for further processing. Fields within these two tables were mapped to a valid SF2 input field name (Table 3). Records with a start date not within the respective year and records missing location or area burned information were removed because they lacked the required descriptive information. Occasionally the same fire may be recorded multiple times. To avoid the duplication of burned area, records were removed from each SIT-209 table where the incident number and the location rounded to three decimal-degrees duplicated a preceding record. Because a different incident number may be used to record the same fire event, a second round of duplicate removals were made where records were dropped as duplicates on unique combinations of the incident name, county, and area fields. In many cases the end date resulted in an event duration beyond the upper quartile for the fire size because the fire could have been reported days or weeks after the event. Very small events with total burned area less than 80.94 hectares are unlikely to actively burn for more than a day and therefore had the end date reset to the day after the start date. Events larger than 80.94 hectares and with a duration greater than 10 days that had a burned area per day rate lower than the first quartile were given a new end date based on the duration calculated from the product of the median area burned per day and the event size. Incident numbers were updated with the record number to create fully unique incident identifiers.

**Table 3:** Mapping of SIT-209 report fields to input name.

INCIDENTS_209_REPORTS Name	SIT209_HISTORY_INCIDENTS Name	SF2 Input Name
REPORT_TO_DATE	CREATED_DATE	report_date
INCIDENT_NUMBER	INCIDENT_NUMBER	incident number
INCIDENT_NAME	INCIDENT_NAME	incident name
DISCOVERY_DATE	DISCOVERY_DATE	start date
POO_STATE_CODE + POO_COUNTY_CODE	POO_STATE_CODE + POO_COUNTY_CODE	county
POO_LATITUDE	POO_LATITUDE	latitude
POO_LONGITUDE	POO_LONGITUDE	longitude
CURR_INCIDENT_AREA	INCIDENT_AREA	area

### 4.2 Configuration of the SF2 system

**Fig. 5:**
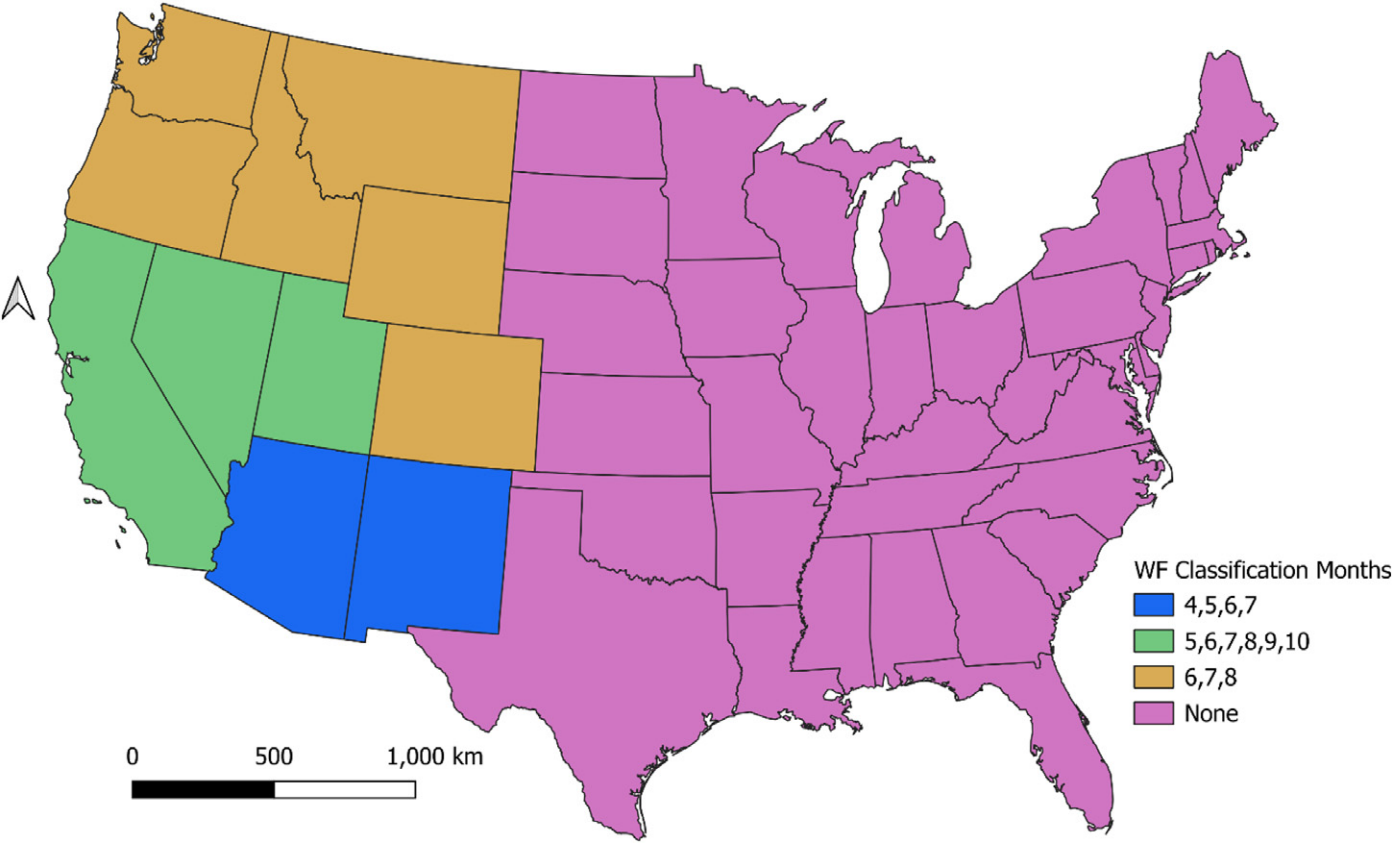
Numerical months where HMS detects are classified as wildfire (WF).

Each annual dataset was imported into the SF2 system using a series of method options that are associated with a data type (e.g., satellite, ground, perimeter) and source format [Table 4]. These options control how SF2 reads and aggregates each input dataset [3]. The fire type for the events within each dataset were characterized using either a field in the dataset (Field), a default value (Default), or a fire type value corresponding to the month and state of the event (Time Period). The Time Period fire type method is used when there is not corresponding fire type information in the dataset, such as with remote sensed fire detects. The Time Period method in SF2 uses a regional climatological approach where a detection is classified as a prescribed fire type except when occuring during the summer in a western state [Fig. 5]. This method is the primary source of uncertainty in the fire type classification process.

**Table 4:** SF2 dataset import configuration.

Dataset Name	Geometry Type	Clump Method	Association Method	Fire Type Method	Ingest Method
HMS	Point	HMS	HMS	Time Period	HMS
GeoMAC	Polygon	GeoMac	GeoMacShapefile	Default	GeoMac
MTBS	Polygon	MTBS	MTBS	Field	MTBS
ICS-209	Point	ICS209	ICS209	Field	ICS209

**Fig. 6:**
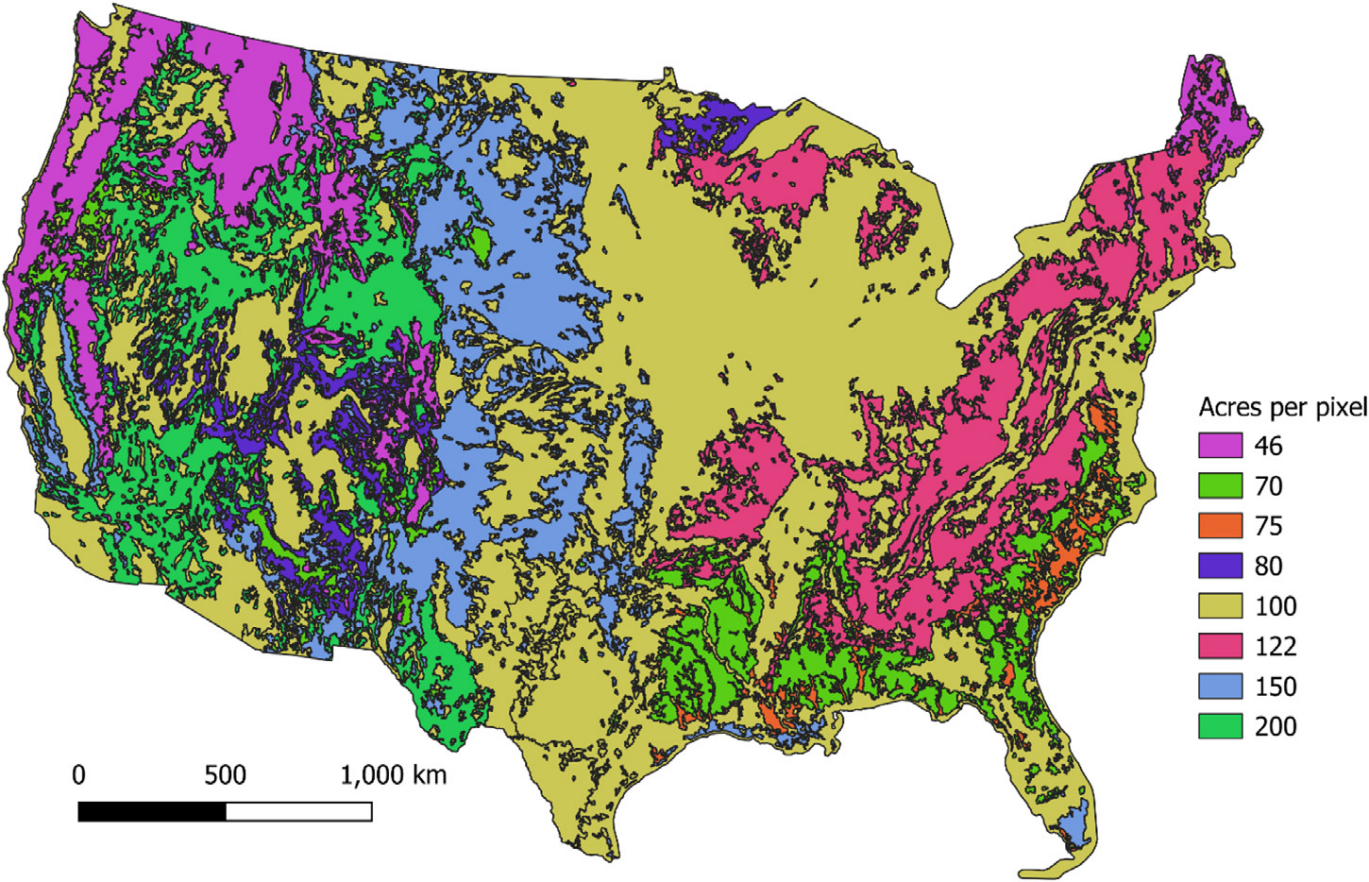
Acres per HMS pixel by vegetation class.

The fire size of HMS detects is not provided and must be assumed. The size of the HMS detects is determined using the average area burned per HMS pixel over broad FCCS vegetation classes (Fig. 6) [11]. Average area per pixel was calculated by overlaying HMS detects over geospatial perimeters for individual fires. The default pixel area values applied in the EQUATES SF2 modelling were originally developed for the 2008 National Emissions Inventory (NEI) [3]. Each HMS detect processed in SF2 is assigned the area per pixel of the underlying vegetation class at the location of the detect [Table 5].

**Table 5:** Assumed acres per HMS pixel by vegetation class.

Vegetation Class Name	General Group	Acres per Pixel
Aspen	Forest	80
Boreal	Forest	100
Closed_Conifer_Forest	Forest	46
Eastern_Deciduous_Forest	Forest	122
Grassland	Grass	150
Juniper	Shrub	80
Open_Conifer_Forests	Forest	70
Other	Mixed	100
Pacific_broadleaved_Forest	Forest	150
Riparian	Forest	75
Savanna	Grass	100
Shrubland	Shrub	200

The combined data sources were geospatially reconciled into a single activity stream containing attributes of each of the input datasets. A set of attribute weights were used to determine which dataset attributes were passed to the output event polygons [Table 6]. Spatial and temporal buffers were used during the reconciliation process to reduce the impact of uncertainty from each data source [Table 7]. The weighting factors and buffer attributes are based on an analysis of the relative strengths and weaknesses for each dataset. For example, geospatial perimeter datasets such as MTBS and GeoMAC provide a more accurate representation of the shape of the fire than the ground data in ICS-209, therefore these datasets receive a higher weighting in the shape category. The weightings and buffers are consistent with the relative weightings and buffers used in the development of the 2017 National Emissions Inventory wildland fire activity data [12].

**Table 6:** Reconciliation weighting factors.

Dataset Name	Location	Size	Shape	Growth	Name	Fire Type
HMS	0.9	0.3	0.5	0.6	0.1	0.2
GeoMAC	0.9	0.85	0.8	0.55	0.81	0.7
MTBS	0.99	0.9	0.9	0.1	0.7	0.4
ICS-209	0.5	0.7	0.2	0.3	0.8	0.9

**Table 7:** Uncertainty buffer values.

Dataset Name	Location	Start Date	End Date
HMS	4.0 km	6 days	5 days
GeoMAC	1.0 km	3 days	6 days
MTBS	0.5 km	5 days	15 days
ICS-209	4.0 km	3 days	5 days

### 4.3 Event polygon dataset cleaning

Annual event reconciliation streams were extracted as geospatial polygons from the event table in the SF2 postgres database using the pgsql2shp tool [13]. Issues in the reconciliation process or with the underlying activity datasets occasionally resulted in duplicate and low confidence wildland fire events. Low confidence scores primarily occur when a fire event from ICS-209 is not corroborated by an event in another dataset. Fire events with the same event name, approximately the same size, ignition in the same month, and within the same state were removed if manually confirmed as a duplicate based on these attributes. Wildfire events over 404.686 hectares in size with a low confidence value (confidence < 0.92) were compared against the input activity data and contemporaneous third-party fire information sources to verify location, size, and ignition date. Fires with low confidence values that could not be independently verified were removed from the shapefile. A common source of both duplicate and low confidence fires was an inexact location defined in the ICS-209 dataset.

## Limitations

Fire event size, location, duration, type association, and naming are limited by the quality and consistency of the underlying activity datasets from 2004–2017. The events from the EQUATES 2002-2003 fires are not included in the geopackage because the activity available in those years necessitated processing methods without geospatial information. Wildland fire activity for the 2018-2019 EQUATES years are not currently included in the dataset because they were processed with software and datasets not described in this paper. Activity datasets were retrieved with information on all 50 U.S. states but only the conterminous 48 U.S. states were included in this processing. Classification of the type and size of HMS-only events are limited by the assumptions in processing HMS detects as fire activity. The quality and resolution of the landcover used for creating the HMS wildland subset may result in fire events located outside of wildland areas and the CONUS.

## Ethics Statement

This research meets the ethical requirements for publication in Data in Brief. This work does not involve studies with animals and humans, or data collected from social media platforms.

## CRediT authorship contribution statement

**James Beidler:** Methodology, Formal analysis, Data curation, Writing – original draft, Visualization. **George Pouliot:** Project administration, Data curation, Writing – review & editing. **Kristen Foley:** Project administration, Methodology, Writing – review & editing.

## Data Availability

Wildland fire event data for the EQUATES project – 1/1/2005 - 12/31/2017 CONUS (Original data) (UNC Dataverse).
